# Variation in Lifestyle-Related Behavior Among Obese Indian Patients With Non-alcoholic Fatty Liver Disease

**DOI:** 10.3389/fnut.2021.655032

**Published:** 2021-04-14

**Authors:** Charu Arora, Anita Malhotra, Piyush Ranjan, Naval K. Vikram, S. N. Dwivedi, Namrata Singh, Shalimar, Vishwajeet Singh

**Affiliations:** ^1^Department of Home Science, University of Delhi, New Delhi, India; ^2^Department of Home Science, Lakshmibai College, University of Delhi, New Delhi, India; ^3^Department of Medicine, All India Institute of Medical Sciences, New Delhi, India; ^4^Department of Biostatistics, All India Institute of Medical Sciences, New Delhi, India; ^5^Department of Gastroenterology and Human Nutrition, All India Institute of Medical Sciences, New Delhi, India; ^6^Department of Geriatric Medicine, All India Institute of Medical Sciences, New Delhi, India

**Keywords:** NAFLD, obese, diet, physical activity, lifestyle, variation

## Abstract

Lifestyle modification is the mainstay of treatment in Non-Alcoholic Fatty Liver Disease (NAFLD). Published Indian data on the diet and lifestyle of Indian NAFLD patients is scarce. This study explored variation in lifestyle-related behavior among obese patients with NAFLD. Ultrasonography (USG) diagnosed obese NAFLD patients (*n* = 140) were assessed for dietary intake [1-day 24 hours recall, food-frequency questionnaire (FFQ)] and physical activity (PA) [Global Physical Activity Questionnaire (GPAQ)]. Diet quality score (0–30) and physical activity (PA) levels were used to study variation in lifestyle and assess the effect of lifestyle on the severity of NAFLD. Compared to the recommendation, calorie consumption was 25.2% higher in nearly half (53.6%) of the subjects and mean macronutrient intake was imbalanced (60.3% carbohydrates, 12.4% protein, 25.5% fats). Variation was seen in terms of diet quality—good (3%), moderate (54.3%), or poor (43.5%) and intensity of PA—high (15%), moderate (47.9%), or low (37.1%). No patient had a combination of high PA and good diet quality within all grades of NAFLD. Our study suggests wide variation in lifestyles of obese patients with NAFLD and need for a more flexible and individualized modification of their diet and PA.

## Introduction

Non Alcoholic Fatty Liver Disease (NAFLD) is a major cause of liver disease and is strongly linked to a poor lifestyle. As high as 60% of individuals are affected by NAFLD and metabolic syndrome in the developed and developing countries (1). NAFLD is widely prevalent (9–35%) in urban as well as rural areas of India (2). Though not imminently fatal itself, it is associated with several co-morbidities like hypertension, atherosclerosis, heart disease, diabetes, dyslipidemia (3). Untreated NAFLD may progress to cirrhosis and hepato-cellular carcinoma (4).

Currently, lifestyle modification (leading to 5–10% weight reduction) remains the cornerstone of NAFLD treatment in patients. However, there is little consensus on particular diet options and strategies that may be most effective in the treatment of NAFLD (5). The current conventional wisdom is that obese patients with NAFLD are eating too many calories and exercising too little (6). As a result, dietitians and physicians generally practice the “one size fits all” approach to weight loss involving universal “calorie restriction” and “increase in exercise” prescription to all obese patients with NAFLD. Both of these lifestyle modifications are limited by sustainability owing to factors such as patient motivation and difficulty with adherence (7, 8). Only 5% patients achieve the 10% body weight reduction. Subsequently the compliance to lifestyle modification drops and the lost weight is regained soon (9). This possibly happens due to the lack of understanding of the variation in the lifestyle of obese patients with NAFLD and therefore a lack of individualized modification of each NAFLD patients' lifestyle.

Published Indian data on the dietary and lifestyle of Indian obese patients with NAFLD is scarce. We hypothesized that not all obese Indian patients with NAFLD are over-nourished and underactive, and there exists considerable variation in their lifestyles. The understanding of this variation in the lifestyle of obese patients with NAFLD, will help us identify NAFLD patients who need lifestyle intervention at an early stage and will also help in designing targeted and judicious lifestyle intervention programs that are more personalized, effective, and sustainable, especially for the Indian population.

Hence, this study was conducted with the aim to explore the variation in the lifestyles of obese Indian patients with NAFLD in terms of their diet and physical activity (PA) profile.

## Patients and Methods

Data was collected in Department of Medicine and Gastroenterology at a tertiary-care center in India, between July 2018 and December 2019 for an ongoing preliminary phase II trial to assess the efficacy of intensive dietary counseling in obese Indian patients with NAFLD (CTRI/2018/04/013179) and approved by Institute Ethics Committee (IEC434/04.08.2017). In the absence of any data on the efficacy of dietary counseling in Indian patients with NAFLD, a sample size of 140 obese patients with NAFLD (70 in control arm and 70 in the intervention arm) was decided for the preliminary phase II trial keeping in mind the availability of obese NAFLD patients in the department, expected drop out of 20% from the intervention and availability of resources to carry out the intensive dietary counseling sessions. This study reports the findings of data that was analyzed as part of a secondary objective of the trial. Written informed consent was obtained from participants before enrolment. A total of 315 patients with ultrasonography (USG) diagnosed NAFLD were screened and patients aged 18–60 years, Body Mass Index (BMI) between 25 and 39.9 kg/m^2^ and willing to give informed consent were included. Patients with alcohol intake >30 g/day (males) or 20 g/day (females), pregnant and lactating women, patients with uncontrolled diabetes (HbA1c >7%), hypothyroidism (TSH >5.0 μIU/mL), diagnosed psychiatric illness, history of recent long term steroid usage, or any significant weight changes in past three months were excluded.

### Dietary Assessment

Dietary data was collected using 1 day 24 hours dietary recall and analyzed for energy and macronutrient intake using software (“DietCal” version 10.0; Profound Tech Solution based on values from Nutritive Value of Indian Foods, 2017) (10). All the participants were interviewed by a trained clinical nutritionist. Wherever possible information regarding the weights of raw ingredients used in different preparations was obtained to get an idea about the quantities of foods consumed and proportions of different ingredients used. The subjects were asked to indicate sizes such as big, medium and small for fruits and chapatis. The consistency of vegetable and pulse preparations was also asked.

A semi quantitative food frequency questionnaire (FFQ) was used to gather information about the dietary diversity, intake of high fat, sugar, salt (HFSS) products, and sugar-sweetened beverages (SSBs). This data was used to calculate the diet quality score (0–30) of each patient (Supplementary Table 1). Diet quality was clubbed into three categories, namely—poor (0–14), moderate (15–22), and good (23–30).

### Physical Activity Assessment

PA pattern was assessed using the 16 item Global Physical Activity Questionnaire (GPAQ) Version 2. It covers PA involvement at work, while traveling to and from places and while doing recreational activities as well as sedentary behavior. PA levels were categorized into low, moderate, and high according to reported frequency and duration of PA.

### Statistical Analysis

The quantitative and qualitative variables are described using means (SD) and frequency (%), respectively. Association between diet quality score, PA, and severity of NAFLD was assessed using chi square and Fisher's exact test. A *p* value < 5% was considered statistically significant.

## Results

One hundred and forty obese patients (as per the Asia pacific criteria, obesity is defined as those having BMI ≥25.0 kg/m^2^) (11) with NAFLD were enrolled in the study. General characteristics, anthropometric details, biochemical parameters, radiological characteristics, NAFLD related symptoms, and PA levels of the study participants are summarized in Table 1. Our study population comprised of 58 (41.43%) females with a mean age of 41.7 ± 10 years. Of the 140 participants, 126 (90%) were married; 75 (53.6%) had education level of graduate and above and 56 (40%) were unemployed. Out of 58 females, 45 (77.5%) were housewives. More than half the participants (59.3%) belonged to upper socio economic status as per the modified Kuppuswamy index, 2019 (12). No NAFLD related symptom was reported in 42 (30%) patients. Severe acidity (41.8%), abdominal fullness (30.7%), mild abdominal pain (27.9%), and fatigue (24.3%) were some other symptoms that were causing discomfort to NAFLD patients. Mean BMI and waist circumference in our study participants was 30.32 ± 4.09 kg/m^2^ and 103.68 ±11.53 cm respectively. Lipid profile, glycemic profile and liver enzymes of the patients have been reported in Table 1. More than half the patients (51.4%) belonged to the mild fatty liver category with a USG grade 1, followed by 63 (45%) patients with grade 2/moderate fatty liver and 5 (3.6%) patients in grade 3/severe fatty liver. The mean Controlled Attenuation Parameter (CAP) value depicting liver fat of the patients was 330.10 ± 33.95 dB/m.

**Table 1:** Demographic, anthropometric, biochemical, radiological characteristics, NAFLD related symptoms and physical activity level of the study participants.

**Variable**	***n* (%)**
**Age (years)** Mean (SD)	41.7 (10.0)
**Female**	59 (42.1)
**Anthropometry and body composition**	
BMI (kg/m^2^) Mean (SD)	30.3 (4.0)
25–29.9	76 (54.3)
30–34.9	45 (32.1)
35–39.9	19 (13.6)
Waist circumference (cm) Mean (SD)	103.7 (11.53)
Hip circumference (cm) Mean (SD)	102.8 (10.34)
Body Fat percentage Mean (SD)	34.5 (7.87)
Body protein percentage Mean (SD)	13.4 (1.95)
**Biochemical parameters Mean (SD)**	
Fasting blood glucose (mg/dL)	98.7 (14.3)
AST (U/L)	39.2 (23.6)
ALT (U/L)	54.9 (35.5)
Alkaline phosphatase (IU/L)	209.8 (96.9)
Total cholesterol (mg/dL)	187.9 (34.8)
HDL-c (mg/dL)	40.38 (8.24)
LDL- c (mg/dL)	126.5 (36.3)
Triglycerides(mg/dL)	167.8 (78.6)
**Grade of fatty liver^∧^**	
Grade 1 (mild)	72 (51.4)
Grade 2 (moderate)	63 (45.0)
Grade 3 (severe)	5 (3.6)
**Fibroscan**
CAP (dB/m) Mean (SD)	330.10 (33.95)
LSM (kPa) Mean (SD)	7.2 (1.5)
**NAFLD related symptoms**	
Severe bloating and indigestion	68 (48.6)
Severe acidity/gastritis	58 (41.4)
Mild abdominal pain	39 (27.9)
Abdominal fullness	43 (30.7)
Extreme fatigue	34 (24.3)
Asymptomatic	42 (30.0)
**Physical activity level^#^**	
Low	52 (37.1)
Moderate	67 (47.9)
High	21 (15.0)
**Mean sedentary minutes/day** Mean (SD)	637.31 (155.38)

# *Calculated as per GPAQ V 2.0*.

∧ *by USG*.

*CAP, Controlled Attentuated Parameter; LSM, Liver Stiffness Measurement*.

Macronutrient intake and dietary diversity of the study participants in terms of consumption of various food groups along with the intake of processed food items has been explained in Tables 2, 3 respectively. Calorie intake of our patients ranged from 740 to 2,800 kcal/day. There is lack of NAFLD specific dietary guidelines, especially for Asian patients. As per the dietary recommendations for NAFLD obese patients suggested by Kargulewicz et al. (13), the calorie recommendation for men and women is 1200–1600 kcal and 1000–1200 kcal per day, respectively. When compared to this recommendation, 37 out of 81 (45.7%) men were consuming a hyper caloric diet (more than 1,600 kcal) and 40 out of 59 (67.8%) women were consuming hyper caloric diet (more than 1,200 kcal per day). The calorie consumption of each patient was compared with their gender specific recommendation and percentage difference was calculated. The mean of difference was also computed, by which calorie consumption was found to be 25.2% higher in majority (53.6%) of the patients. Variability was observed in the diets which were predominantly loaded with carbohydrates (60% as against the recommendation of 40–50% carbohydrates for NAFLD patients). High carbohydrate consumption was seen in 95% of patients. Protein intake was found inadequate (12% as against the recommendation of 15–20% of protein in the daily diets of NAFLD patients), and 90% patients were consuming a low protein diet. The mean fat intake of the patients (25.4%) was within the prescribed limit of <30% of daily calories. However, fat consumption was found to be higher than the recommended intake in almost 25% patients.

**Table 2:** Energy and macronutrient intake and variation in diets.

**Total energy and macronutrient intake**		
**Energy/macronutrient**	**Mean intake Mean (SD)**	Recommended intake^*^
Total energy (kcal)	**Male:** 1,629 (398) **Female:** 1,346 (384) **Clubbed:** 1,509 (384)	**Male:** 1,200–1,600 **Female:** 1,000–1,200
Total carbohydrate (g)	227.7(58.0)	
Total protein (g)	46.8(12.8)	
Total fat (g)	42.8(18.5)	
**Macronutrient intake in terms of percent energy**		
**Macronutrient**	**Intake (% energy)**	Recommended intake^*^ (% energy)
Total carbohydrate	60.3	45–50
Total protein	12.4	15–20
Total fat	25.5	<30
**Distribution of subjects by variation in diets**		
**Variation in diets**	***n***	**%**
Hypo-caloric Normo-caloric Hyper-caloric	20 45 75	14.3 32.2 53.6
High carbohydrate	133	95.0
Low protein	126	90.0
High fat	75	53.6

* *Dietary recommendations for subjects with obesity and NAFLD (13)*.

**Table 3:** Dietary diversity and intake of HFSS and SSB among study subjects.

**Dietary intake**	***n* (%)**
**Coffee intake (cups/day)**	
More than 3 cups	1 (00.7)
1–2 cups	15 (10.7)
None	124 (88.6)
**Type of milk used**	
Double toned	4 (2.9)
Toned/cow milk	50 (35.7)
Full cream (packet)/buffalo	84 (60.0)
No milk consumption	2 (1.4)
**Fruit consumption**	
Daily	19 (13.6)
2–3 times a week	57 (40.7)
<2–3 times a week	64 (45.7)
**Vegetable consumption (including salads)**	
2–3 times a day	85 (60.7)
Once daily	40 (28.6)
2–3 times a week	15 (10.7)
**Pulses/legumes consumption**	
Twice a day	24 (17.1)
Once a day	91 (65.0)
≤2–3 times a week	25 (17.9)
**Nuts and oilseeds consumption**	
Daily	15 (10.7)
2–3 times a week	15 (10.7)
Once a week	19 (13.6)
Once a fortnight or less	91 (65.0)
**Meat and meat product consumption**	
Daily	17 (12.1)
2–3 times a week	23 (16.4)
Once a week	21 (15.0)
Once a fortnight	10 (07.1)
Once a Month	15(10.7)
Never	54 (38.6)
**Added sugar/jaggery/honey (teaspoons/day)**	
1–2	32 (22.9)
3–5	83 (59.3)
>6	25 (17.9)
**Sugar sweetened beverages/juices consumption**	
Once a fortnight or less	57 (40.7)
Once a week	38 (27.1)
2–3 times a week	31 (22.1)
Daily	14 (10.0)
**Sweets consumption (*****Ladoo, Halwa, Burfi)***	
Once a fortnight or less	45 (32.1)
Once a week	50 (35.7)
2–3 times a week	28 (18.7)
Daily	17 (12.1)
**Fast foods consumption (pizza, burger, momos)**	
Once a fortnight or less	69 (49.3)
Once a week	35 (25.0)
2-3 times a week	29 (20.7)
Daily	7 (5.0)
**Bakery products consumption (biscuits, cookies, cakes)**	
Once a fortnight or less	14 (10.0)
Once a week or less	5 (3.6)
2–3 times a week	44 (31.4)
Daily	77 (55.0)
**Fried Indian savory snacks consumption (*****samosa, pakoda, bhujia, mathri*****)**	
Once a fortnight or less	51 (36.4)
Once a week	27 (19.3)
2–3 times a week	46 (32.9)
Daily	16 (11.4)

The food frequency data revealed that the daily intake of fruit and vegetable was low as only 19 (13.6%) patients reported consuming atleast one regular serving of fruit and 85 (60.7%) patinets reported consuming a minimum of two to three servings of vegetables. The number of vegetarians was 54 (38.6%) and occasional meat eaters was 46 (32.8%) which was substantial, yet the intake of plant protein was found to be inadequate with only 24 (17.1%) participants consuming pulses two times a day. Nuts and oilseed consumption was reported by only 15 (10.7%) patients. Full cream milk was the choice of dairy for majority 84 (60%) patients. Sugar consumtion was moderately high as 83 (59.3%) participants were reported to be consuming three to five teaspoons while 25 (17%) patients consumed 6 teaspoons or more of sugar, honey, and/or jaggery daily. Intake of sugar sweetened beverages and fruit juices (fresh/packaged) up to three times a week was reported by one third (32%) of the participants. Fast food consumption on a daily basis was reported by one fourth of the patients. More than half of them 77 (55.0%) were regularly consuming bakery products while close to half, 62 (44.3%) patients consumed fried Indian savory snacks on a frequent basis.

Nearly half, 67 (47.9%) study participants were involved in moderate level physical activity, while 52 (37.1%) and 21 (15%) patinets were engaged, respectively, in low and high levels of physical activity per day (Table 1). The mean sitting time per day was 9–10 h per day, and was significantly higher for men (637.3 ± 155.4 min/day) as compared to women (523.4 ± 116.1 min/day) (*p* = 0.000).

To explore the variation in the lifestyle of our obese patients with severity of NAFLD, we developed a matrix based on their grade of fatty liver, diet quality and PA level (Table 4). This matrix gives a clear picture of how diet and physical activity levels vary among the study participants with the change in severity of fatty liver. Overall, the diet quality was found to poor (43%) or moderate (54%), irrespective of the severity. No significant association was found between grade of fatty liver and diet quality (*p* = 0.197) or between grade of fatty liver with level of physical activity (*p* = 0.615). Also, no significant association was reported between diet quality and PA level (*p* = 0.138). None of the patients had a combination of high PA and good diet quality. A moderate level of PA with moderate diet quality was found in 29% of patients. Another 37% of them had low PA with either poor (50%), or moderate (50%) diet quality.

**Table 4:** Matrix depicting variation in diet quality and intensity of physical activity as per severity of NAFLD.

**Grade of fatty liver (Severity of NAFLD)**	***n* (%)**	**Components of lifestyle**			
		**Intensity of physical activity**	***n* (%)**	**Quality of diet**	***n* (%)**
Grade 1 (Mild fatty liver)	72 (51.4)	Low	24 (33.3)	Poor	10 (41.7)
				Moderate	14 (58.3)
				Good	0
		Medium	37 (51.4)	Poor	13 (35.1)
				Moderate	21 (56.8)
				Good	3 (8.1)
		High	11 (15.3)	Poor	6 (90.9)
				Moderate	5 (45.5)
				Good	0
Grade 2 and 3 (Moderate to	68 (48.6)	Low	28 (41.2)	Poor	16 (57.1)
severe fatty liver)				Moderate	12 (42.9)
				Good	0
		Medium	30 (44.1)	Poor	10 (33.3)
				Moderate	20 (66.7)
				Good	0
		High	10 (14.7)	Poor	6 (60.0)
				Moderate	4 (40.0)
				Good	0

## Discussion

Our study has tried to explore in detail the diet as well as the physical activity status of 140 USG diagnosed, obese NAFLD patients, visiting a tertiary care center in North India. Although, we did not find any significant association between severity of NAFLD and lifestyle among our patients, our study supports our hypothesis that there is a considerable variation in energy and macronutrient intake, diet quality and PA of obese patients with NAFLD. The range of caloric intake of our patients varied from as low as 780 kal to as high as 2,800 kcal. The average diet quantity (mean caloric intake of 1,509 kcal/day) was moderately high in terms of calories and carbohydrates, but low in proteins. In terms of quality, the diets ranked from poor to moderate. None of the participants exhibited good diet quality or good PA. Low to moderate PA level was seen in 85% patients.

With no approved drug therapy, weight loss remains the background treatment for patients with NAFLD and obesity (14). A 5% reduction in BMI can be accompanied by 25% reduction in liver fat (15). Diet and PA are complimentary yet independent components of weight loss. It is assumed that all obese patients with NAFLD are over-nourished and under-active (6). Our study exhibits considerable variation in lifestyle adequacy when diet and PA components were clubbed together. A wide variation in the caloric intake of NAFLD patients (1,009–5,594 kcal/day) has also been reported in a study carried out on 55 adults with NAFLD in Germany (16).

Diets were hyper-caloric for half (50%) of the patients, but the surplus calories did not go beyond 25% of the recommended intake. Other half (50%) of the patients were consuming low to normal calories as compared to the recommendation. Patients with NAFLD are often asymptomatic. When asked to restrict calories for treatment of NAFLD, these patients are often left confused, as many of them are already eating within the calorie limit. Weight gain has been typically framed as a problem of excess caloric intake, but what is often ignored is that the quality of diet is also associated with long-term weight gain (17). Irrespective of caloric intake, half of our participants had imbalanced macronutrient distribution, with high carbohydrates, low protein, and poor diet quality characterized by low dietary diversity and high consumption of HFSS foods. Given that NAFLD is also present in non-obese individuals, it is evident that NAFLD is not simply a result of excessive energy intake, but can be associated with quality of diet (18). Browning et al. randomized 18 individuals with NAFLD into two groups following either a low-carbohydrate or a low-fat diet with similar calories, for 2 weeks, each with a similar goal of 5% weight loss. Despite iso-caloric intake and similar weight loss, individuals on the low-carbohydrate diet experienced a nearly 60% reduction in hepatic fat, compared with only a 25% reduction in hepatic fat for individuals on the low-fat diet. This data explains that calorie for calorie, weight for weight, different foods have a differential effect on the metabolism and body function in NAFLD. The diet quality and composition not only influences the development and progression of NAFLD (19) but may also alter energy expenditure during weight loss maintenance (20).

Despite moderate calorie consumption, our participants were frequently consuming high fat, salt, and sugar (HFSS) food items. Our findings are very similar to a recent dietary survey conducted on 98 NAFLD patients in Pakistan (21).

Lifestyle modification through increased physical activity is beneficial in patients with NAFLD. Although weight loss has been shown to produce improvement in biochemical and histological markers of NAFLD, exercise might improve hepatic steatosis and steatohepatitis even in the absence of major weight loss. Though, the overall PA level of NAFLD patients in our study was low to moderate, these levels are higher than those reported by a 3-year old study conducted at the same locale using GPAQ 2.0; high (15 vs. 5%), moderate (47 vs. 39.3%) and low (37 vs. 55.62%) (22), suggesting a changing trend in PA of NAFLD patients seeking treatment at a tertiary care hospital. There is also evidence to suggest that PA is less important for weight loss because calories burned by PA may be lower than the calorie deficit achieved by dietary restriction (23). However, PA can reduce weight and hepatic fat in a gradual manner and it may be promoted in patients who are non-adherent to dietary restrictions or it may be combined with diet to improve weight loss. Our study brings to light that overweight and obese Indian NAFLD patients have as high as 9–10 h of sitting per day. High sitting times (more than 8 h/day) compared to low sitting times (<4 h/d) almost double the risk of developing type 2 diabetes and increase the incidence of cancer and CVD by 10–20% (24). Targeting a reduction in sedentary time, without increasing physical activity, might also improve NAFLD, though more research is still needed in this area (7).

Only one fifth of participants in our study had a combination of poor diet and low PA. Majority of the participants were dispersed in the middle range with either lifestyle component (diet or PA) requiring minor to major improvement. Our findings highlight the importance of identifying specific patients who will benefit from diet modification (diet diversity, diet quality), increase in PA (during work, leisure, travel, and sedentary minutes per day) or a combination of both to achieve long term successful clinical outcomes, thus emphasizing need for a flexible and individualized approach to treatment. Focus needs to shift from just diet quantity toward diet quality along with emphasis on increase in PA during work, leisure, and travel. Need of the hour is to work on an individualized approach focussing on detailed study of NAFLD patient's diet, physical activity, and lifestyle profile before writing the lifestyle change prescription, to ensure better compliance, and more successful treatment outcomes.

Our study is arguably limited by several aspects of design and analysis. Based on our small sample size, our results may not be generalizable to all obese patients with NAFLD. Hence, the importance of this study is restricted to hypothesis generation only. In terms of our 24 h dietary recall, FFQ, and GPAQ data, we cannot rule out recall errors and bias. However, we used cognitive interviewing techniques (memory cues and think aloud techniques) to improve recall. Nevertheless, the strength lies in the findings which pave way for larger studies assessing the variation in the lifestyle of obese patients with NAFLD. Future researchers may also focus on the presence of xenobiotics in the diet that may inactivate anti-aging genes and supersede the beneficial effects of diet and exercise on NAFLD.

In summary, this study demonstrates a wide variation in lifestyles of obese patients with NAFLD. In the absence of a clear consensus on the best dietary and lifestyle strategies to treat NAFLD and lack of NAFLD specific dietary guidelines, especially for Asian patients, it is crucial that health care professionals treat each patient as a unique case and assess in depth, the components of his/her lifestyle before writing a prescription for lifestyle change. “Calorie quality” needs to be brought in focus along with “calorie counting” within a personalized diet chart, along with intensive counseling to encourage patients to engage in regular PA based on their age, physical status, time availability, and family support. Dietitians in liver clinics should lead as lifestyle interventionists and design individualized interventions for their patients with NAFLD, keeping in mind the variation that exists in the lifestyle of patients with NAFLD.

## Data Availability Statement

The raw data supporting the conclusions of this article will be made available by the authors, without undue reservation.

## Ethics Statement

The studies involving human participants were reviewed and approved by Institute Ethics Committee, All India Institute of Medical Sciences, New Delhi. The patients/participants provided their written informed consent to participate in this study.

## Author Contributions

CA: data collection and writing of the manuscript. AM: concept and study design, data interpretation, revision, critical review, and finalization of the manuscript. PR: concept and design, data interpretation, and final approval of manuscript. NV: revision and review of the manuscript. SD: critical review of paper. NS: data collection, analysis, and interpretation of data. S: critical review of paper. VS: statistical analysis and data interpretation. All authors contributed to the article and approved the submitted version.

## Conflict of Interest

The authors declare that the research was conducted in the absence of any commercial or financial relationships that could be construed as a potential conflict of interest.
