# Supplementary material for: Variation in Lifestyle-Related Behavior Among Obese Indian Patients With Non-alcoholic Fatty Liver Disease
Source: Front Nutr. 2021 Apr 14;8:655032. doi: 10.3389/fnut.2021.655032 (PMC8079655; doi:10.3389/fnut.2021.655032)
Supplement: Supplementary file 1 [file Table_1.DOCX]

**Supplementary table 1**: **Scoring criteria to calculate diet quality of the study participants**

| **Criteria** | **Frequency** | **Score** |
| --- | --- | --- |
| 1. Fruit intake | Less than or equal to 2 times a week  3-5 times a week  6-7 times a week | 0  1  2 |
| 1. Vegetable intake | Less than once a day  Once a day  2 or more times/day | 0  1  2 |
| 1. Pulse intake | Less than once a day  Once a day  2 or more times/day | 0  1  2 |
| 1. Dairy intake | Less than or equal to 2 times a week  3-5 times a week  6-7 times a week | 0  1  2 |
| 1. Red meat intake | More than once a week  Once a week  Less than once a week | 0  1  2 |
| 1. Poultry and fish intake | Less than once a week  Once a week  More than once a week | 0  1  2 |
| 1. Nuts and oilseeds intake | Less than or equal to 2 times a week  3-5 times a week  6-7 times a week | 0  1  2 |
| 1. Bakery items intake (Breads/biscuits/cookies) | More than once a week  Once a week  Less than once a week | 0  1  2 |
| 1. Fast food intake (Pizza, burger, noodles, momos etc.) | More than once a week  Once a week  Less than once a week | 0  1  2 |
| 1. Fried food intake (pakodas, samosa, bhujia etc) | More than once a week  Once a week  Less than once a week | 0  1  2 |
| 1. Sweets intake (Halwa, barfi, laddu etc) | More than once a week  Once a week  Less than once a week | 0  1  2 |
| 1. Sugar sweetened beverages/aerated drinks and fruits juice intake | More than once a week  Once a week  Less than once a week | 0  1  2 |
| 1. Preference for low fat dairy | Always  Sometimes  Never | 0  1  2 |
| 1. Preference for whole grains/low GI foods | Always  Sometimes  Never | 0  1  2 |
| 1. Added sugar intake/day | 1-2 teaspoons  3-5 teaspoons  More than 6 teaspoons | 0  1  2 |

Total score: 30

- Good diet: 23-30
- Diet needs improvement: 15-22
- Poor diet: 0-14
